# Robot-Assisted Microsurgery—what does the learning curve look like?

**DOI:** 10.1016/j.jpra.2024.07.009

**Published:** 2024-07-31

**Authors:** Helena Frieberg, Jessica M. Winter, Olof Engström, Daniel Önefäldt, Anna Nilsson, Maria Mani

**Affiliations:** Section of Plastic and Maxillofacial Surgery, Department of Surgical Sciences, Uppsala University, and Department of Plastic and Reconstructive Surgery, Uppsala University Hospital, Uppsala, Uppsala, Sweden

**Keywords:** Microsurgery, Robot-assistance, Anastomosis, Learning curve

## Abstract

**Background:**

The introduction of robotic assistance in surgical practice has led to advancements such as the MUSA-2 robotic system that was designed for microsurgical procedures. Advantages of this system include tremor filtration and motion scaling. Initial studies showed promising results in skill acquisition for robot-assisted microsurgery. This study evaluated the learning curve for microsurgical anastomosis with and without robotic assistance among surgeons of varying experience levels.

**Methods:**

Fifteen surgeons were divided into 3 groups (novice, intermediate, and expert) based on their microsurgical experience. They performed 10 anastomoses by hand and 10 with robotic assistance on synthetic polyvinyl alcohol vessels (diameter of 2 mm) in a laboratory setting. Participants were timed and mistakes such as backwall and leakage were assessed and recorded. Demographic information was collected.

**Results:**

Statistical differences were found in manual anastomosis times between the intermediate and novice groups compared to the experts (p < 0.01). However, no statistical difference was found in the mean time between groups for the robot-assisted anastomoses. *Novice doctors had the steepest learning curve for hand-sewn**anastomosis*. Experts had the fastest completion time at the end of the 10^th^ robotic session, finishing at 14 min, compared to 33 min at the 2^nd^ session. All groups reduced their mean time in half through their 10 robotic sessions.

**Conclusion:**

This study indicated similarities in the learning curves for robot-assisted anastomosis among surgeons with varied experience levels. Experts excelled technically in manual anastomoses, but robot-assistance enabled novice and intermediate surgeons to perform comparably to the experts. Robotic assistance may aid more novice learners in performing microsurgical anastomosis safely at earlier points in their education.

## Background

Innovations in surgical practice through research and technology is at the forefront of plastic surgery; integrating the surgical robot was no exception.^1^ Robotic surgery is widely used in different surgical specialties today. The Da Vinci surgical system (*Intuitive Surgical Inc., Sunnyvale, CA),* initially developed for laparoscopy, is the most commonly used system.^2^, ^3^, ^4^ Advantages of robotic assistance include scaling of the surgeon's motions and elimination of natural tremors, enabling more precise maneuvers in hard-to-reach locations. It can also improve ergonomics, which is increasingly important during long operations. The possibility of increasing precision while decreasing the physical stress of long and demanding procedures has led to an increased interest in robotic assistance within microsurgery and supermicrosurgery in recent years.

Microsurgical procedures have developed alongside the refinement of instruments and improvements in magnification leading to the development of supermicrosurgery, which refers to anastomosis of vessels with diameters between 0.3 and 0.8 mm.^5^ Mentions of lymphedema has seen a significant increase in studies on oncology owing to the concern regarding psychological and physical extremity function.^6^ The possibility of performing anastomoses on a supermicrosurgical level has revolutionized lymphedema treatment through lymphatic venous anastomosis (LVA) surgery,^7^ which has shown promise in early-stage lymphedema regarding ease of progression and is considered safe to perform.^8^

Different automated systems have been tested for microsurgery, such as the Da Vinci surgical system and Zeus robotic surgery system (*Computer Motion, Goleta, California*).^2^, ^3^, ^4^^,^^9^^,^^10^ In a microsurgical context, there are limitations with the Zeus robotic surgery system and Da Vinci surgical system due to the bulky built-in instruments and camera. The instruments are not the same as the refined, specially developed microinstruments used in microsurgery and supermicrosurgery for handling delicate tissues. Other limiting factors are the high cost and cameras in each system lacking the magnification provided by microscopes.^2^^,^^3^^,^^9^ In plastic surgery, robotic use has been cited for transoral reconstruction, muscle and perforator flap harvest, and microsurgery.^1^^,^^11^

The MUSA-2 (*MicroSure, Eindhoven, The Netherlands*) is a new robotic system designed for microsurgery to eliminate some of the limitations of other robotic systems. The engineers at The Eindhoven University of Technology, in cooperation with the microsurgeons at the Maastricht University Medical Centre in the Netherlands constructed a system that allows the surgeon to use the same fine microsurgical instruments and microscope that are used during manual microsurgery. The surgeon controls the instruments using joysticks, which filter tremors, scale down motions, and increase precision.^12^ In recent years, the MUSA-2 has been tested in vitro and in vivo, proving its clinical utility.^13^, ^14^, ^15^ Since the inception of the MUSA-2, additional surgical robots have been developed and dedicated to performing microsurgery, including the Symani surgical system (Medical Microinstruments, S.p.A, Calci, Pisa, Italy) developed in 2019 to overcome the large-scale movements required by the Da Vinci system.^16^^,^^17^

Several factors are of importance when evaluating the learning process, including time required for task completion, quality of the finished product, and the rate of improvement with training, i.e., the learning curve.^18^ The developing center of the MUSA-2 robot found in their initial study that in robot-assisted microsurgery, the learning curve is steeper for robot-assisted anastomosis than that for manual anastomosis and that robot assistance required more time compared to hand sewing, regardless of previous surgical experience.^13^ Other studies have shown that traditional microsurgery training is not a prerequisite to learning robot-assisted microsurgery.^3^^,^^19^^,^^20^

Our center is the first site outside the developing center in the Netherlands to test and use the MUSA-2 robot. Although there have been studies on skill acquisition among surgeons,^21^^,^^22^ the current study examines the learning curve among a more extensive set of learners at different levels of training and practice. This study aimed to evaluate the difference in skill acquisition rate for robotic microsurgery among practitioners with different levels of microsurgical experience. We hypothesize that inexperienced surgeons can achieve higher quality anastomosis at an earlier stage with robotic assistance compared to manual anastomosis methods.

## Materials & methods

Ethical approval was granted by the regional ethics committee in Uppsala (2019-05040). Fifteen medical doctors participated and were divided into 3 groups (5 doctors each) based on their previous experience with microsurgery: Group 1, previously trained microsurgeons (experts); Group 2, surgical residents (intermediate); and Group 3, doctors who had none or <3 months of microsurgical experience (novice). Each participant performed 10 anastomoses by hand and 10 robot-assisted anastomoses. The number of anastomoses was decided based on a previous study, which demonstrated that a learning plateau would be reached after 8-10 anastomoses.^22^ Before performing the first anastomosis using the MUSA-2 robot, 2 different training exercises were completed (a total of 30 min); first, the participants followed 3 lines with the microsurgical instruments to learn to control the robotic system and understand its range limitations. Second, they practiced positioning the needle in the needle holder.

**Figure 1:**
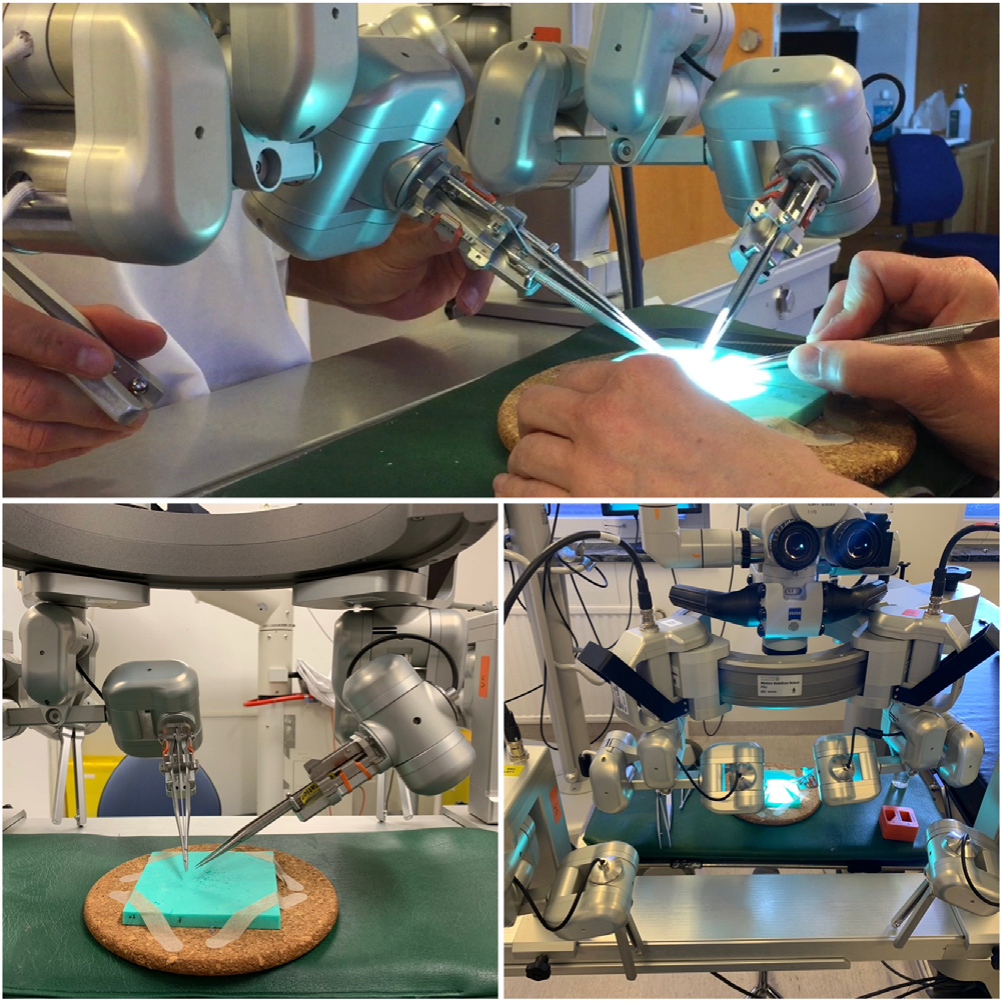
Robotic set-up in the laboratory. Pictures of the robotic set-up from different angles. The upper picture shows one of the participants performing the anastomosis using the joystick to maneuver the instruments. The assistant sits opposite. The left picture shows how the instruments are positioned while suturing. The right picture shows how the microscope is positioned over the robotic arms holding the instruments.

The participants performed 2 anastomoses during each session, 1 manual and 1 using MUSA-2. The sessions always started with manual anastomosis. The sessions were conducted using a Zeiss OPMI PRO Magis S8 surgical microscope in a microsurgery lab. Each participant was instructed on how to change the variable magnification and light, and they chose the set-up that best suited their individual needs. In sessions where only 1 anastomosis was performed, the participants alternated methods between sessions. The days between sessions were calculated and counted separately as days between manual anastomosis and days between anastomosis using MUSA-2. There was a 60-minute time limit for each session. Robotic set-up in the laboratory (Figure 1) demonstrating how the robot is used.

The anastomosis was done on 2 mm diameter polyvinyl alcohol vessels using 9-0 Ethilon sutures. The participants were instructed to perform the anastomosis using 6 single stitches and aim for no leakage. No other instructions were given on how to perform the stitches. During each anastomosis, the participant had an assistant. Throughout the study, there were 5 alternating assistants. While using the MUSA-2, the participant would place the stitch and tie the knots. The primary task for the assistant was to pull the suture through once the participant had placed the throw and cut the threads, as these tasks are larger-scale movements and preferably performed by an assistant. In addition, the participant was helped with whatever they asked for, such as flipping the vessel to suture the back wall. No vascular clamps were used.

The research assistant evaluated each anastomosis for errors; leakage or a back wall stitch, if present, was recorded. Red-colored water was used to assess leakage and flow before the vessel was cut to inspect the anastomosis site for patency. Each participant was given feedback on their performance following each anastomosis.

The data were collected prospectively. Total anastomosis time was measured from when the participant put the needle through the vessel for the first stitch until the threads were cut on the sixth stitch (i.e., the last stitch). Demographic data were collected on the participants.

Simple statistics were performed to calculate the mean time for anastomoses among the groups and total mistakes. Pilot testing resulted in outliers with the risk of heavily skewing the result; therefore, session 1 was discarded from the hand-sewn and robotic groups. The average times from each session were plotted with standard deviation among each group using GraphPad Prism (*version 10.0.2 for macOS, GraphPad Software, Boston, Massachusetts, USA*). Trend lines were determined among each group. The student's *t-*test was used to analyze the average mean times for the second, fifth, and tenth anastomoses to compare the intermediate and novice groups to the expert group. A p-value of <0.05 was considered statistically significant.

## Results

Demographic data (Table 1) included sex, age, handedness, activity level, previous robotic experience, and previous video game experience. Eight females and 7 males participated, varying in age (range 27-65 years) and microsurgical experience. There were 5 participants in each group who each performed 10 sessions. For each group, 1 participant did not finish the last 5 sessions, leading to 4 participants per group for the last 5 sessions. Most participants were right-handed. No participants had previous experience using MUSA-2, and only 3 participants had experience with a robotic device. The experience with video games ranged from none to moderate. Overall, the demographics were similarly matched among the groups aside from age and surgical expertise.

**Table 1:** Demographic data on participants.

*Surgeon*	*Sex*	*Age (years)*	*Activity level*	*Handedness*	*Previous robotic experience*	*Video game experience*
*1.1*	*M*	*54*	*Light*	*Right*	*None*	*1*
*1.2*	*M*	*65*	*Sedentary*	*Right*	*None*	*0*
*1.3*	*F*	*35*	*Light*	*Right*	*None*	*0*
*1.5*	*M*	*39*	*Very Active*	*Right*	*None*	*1*
*1.6*	*M*	*34*	*Moderately*	*Left*	*None*	*0*
*2.1*	*F*	*36*	*Moderately*	*Right*	*Laparoscopic*	*2*
*2.2*	*M*	*34*	*Light*	*Right*	*None*	*1*
*2.3*	*F*	*39*	*Moderately*	*Right*	*None*	*0*
*2.4*	*F*	*36*	*Moderately*	*Right*	*Laparoscopic*	*0*
*2.6*	*F*	*29*	*Light*	*Right*	*None*	*1*
*3.1*	*M*	*30*	*Sedentary*	*Ambidextrous*	*Laparoscopic*	*0*
*3.2*	*F*	*36*	*Light*	*Right*	*None*	*1*
*3.5*	*M*	*28*	*Light*	*Right*	*None*	*2*
*3.7*	*F*	*27*	*Very Active*	*Right*	*None*	*0*
*3.8*	*F*	*29*	*Light*	*Right*	*None*	*0*

Video games: 0 = none,<100 h; 1 = little <2500 h; 2 = moderate <5000 h; 3 = much <13,000 h; 4 = loads >15,000 h.

Activity Level: sedentary (little/no exercise), lightly active (light exercise/sport 1-3 days/w), moderately active (moderate exercise/sports 3-5 days/w, very active (hard exercise/sports 6-7 days/w, 5 = extra active (very hard exercise/sports & physical job or 2x per day training).

**Figure 2:**
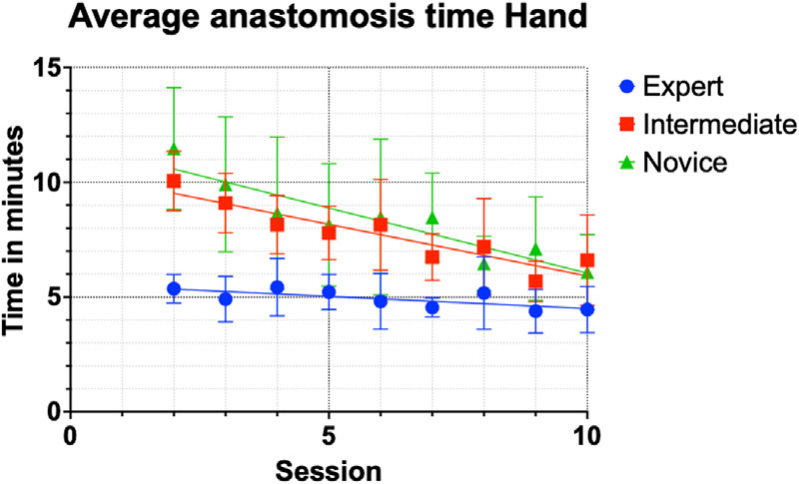
Hand-sewn anastomoses scatter plot graph, average time per session with trendline, and standard deviation. Hand anastomoses, R-squared, and coefficient: expert 0.5389 and −0.1064, intermediate 0.8475, and −0.4503, novice 0.8546 and −0.5672. At the beginning of the study, the expert group was significantly faster in performing hand-sewn anastomoses, but after 10 sessions, no significant differences were found between the groups.

The hand-sewn anastomoses statistics were calculated for the second, fifth, and tenth sessions between the groups. During session 2, the mean time required to complete 1 anastomosis manually was significantly longer in the intermediate (p-value = 0.0001) and novice groups (p-value = 0.001) versus the expert group. In session 5, the time differences were significantly longer for the intermediate (p-value = 0.003) and novice groups (p-value = 0.047) versus the expert group. At the final session, no significant differences were found in the mean duration between the experts and intermediate groups (p-value = 0.085) or between expert and novice groups (p-value = 0.126; Table 2). Overall mean time decreased during each group session (Figure 2), with the intermediate and novice groups exhibiting the steepest learning curve. Examples of the manual procedure performed in each group are demonstrated in attached videos 1-6.

**Table 2:** Average time for hand-sewn anastomoses in min; mean time (standard deviation).

Session	2^nd^	5^th^	10^th^
Expert	5:25 (0:52)	5:21 (0:49)	4:35 (1:03)
Intermediate	10:14 (1:11)*	8:03 (1:10)*	6:50 (1:53)
Novice	11:39 (2:38)*	8:22 (2:44)⁎⁎	6:17 (1:36)

Caption: 5 surgeons per group (total n = 15) average (S.D.) time in min and s to complete 1 anastomosis by hand after the second, fifth, and tenth sessions.

⁎ p < 0.01.

⁎⁎ p < 0.05.

**Figure 3:**
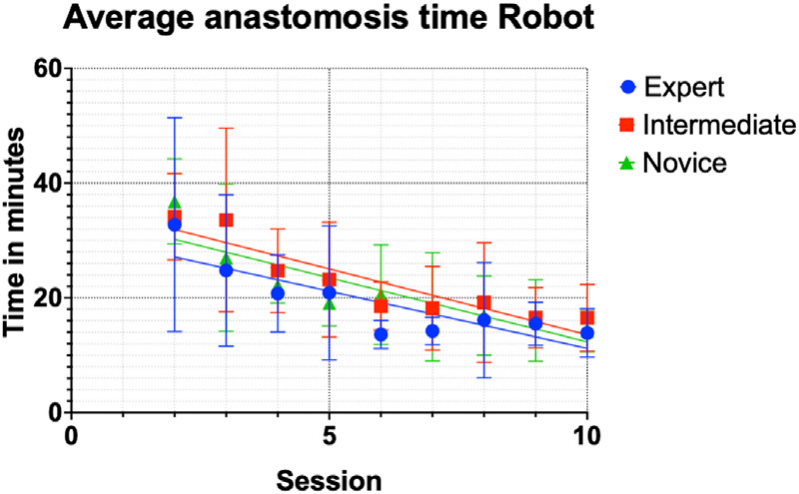
Robot-assisted anastomoses scatter plot graph, average time per session with trendline, and standard deviation. Robot anastomoses, R-squared, and coefficient: expert 0.7241 and −1.990, intermediate 0.8324, and −2.295, novice 0.7804 and −2.232. Throughout the study, the mean times for robot-assisted anastomoses were similar between the groups.

The robotic session statistics were calculated for the second, fifth, and tenth sessions between the groups. Results in the robot group showed no differences in mean time between the expert and intermediate groups or between the expert and novice groups at any point (Table 3); all p-values are >0.05. As seen in the hand-sewn group, the times decreased with increasing sessions in all the groups (Figure 3). Interestingly, the trends were similar among all participants. Experts had the fastest times by the tenth session. Examples of the procedure performed using the robot in each group are demonstrated in the attached videos 7-12.

**Table 3:** Average time for robotic-assisted anastomoses in min; mean time (standard deviation).

Session	2^nd^	5^th^	10^th^
Expert	33:08 (18:49)	21:04 (11:37)	13:58 (04:06)
Intermediate	34:24 (07:27)	23:19 (10:00)	16:43 (05:41)
Novice	36:59 (07:20)	19:16 (03:57)	14:07 (03:50)

Caption: 5 surgeons per group (total n = 15) average (S.D.) time in min and s to complete 1 anastomosis with robot after the second, fifth, and tenth sessions.

Errors were tracked and recorded (Table 4) following each anastomosis. There was a general trend of fewer mistakes in the expert group than in the intermediate or novice groups. The expert and intermediate groups had more errors when performing robotic anastomoses than when sewing by hand. The robot anastomoses carried a lower error rate for the novice group than for manual anastomoses. In general, there was a trend of fewer mistakes among all groups as the sessions progressed. Although the expert group had longer times in the early sessions, there were fewer errors (Figure 3 and Table 4).

**Table 4:** Total errors represented as a percentage of the total possible maximum errors among the groups.

		Session 2-4	Session 5-7	Session 8-10
Expert	Hand	0 (0%)	2 (7.8%)	1 (4.2%)
	Robot	2 (6.7%)	1 (3.8%)	2 (8.3%)
Intermediate	Hand	4 (13.3%)	2 (7.8%)	1 (4.2%)
	Robot	5 (16.7%)	2 (7.8%)	1 (4.2%)
Novice	Hand	7 (23.3%)	5 (19.2%)	3 (12.5%)
	Robot	7 (23.3%)	3 (11.5%)	4 (16.7%)
Maximum		30	26	24

## Discussion

The current study shows that the learning curve for robotic-assisted microsurgery is similar among learners at various levels of experience, managing the technique faster with each session and with preserved quality, which is in line with earlier studies. A previous study by Karamanoukian et al. included 3 microsurgeons and 5 residents performing 80 anastomosis with and without a robot (each performing 5 manually and 5 with the robotic assistance) and found no difference in mean time or errors over the 5 sessions.^19^ Similar to our findings, a blinded evaluation study performed on 10 surgeons with varying levels of experience revealed that previous microsurgical experience resulted in faster skill acquisition with the robot.^20^ However, we found that surgeons with varying degree of experience, similar to the findings from other assessment models, could improve their skills using the robot.^18^ Robotics used in laparoscopic surgery have been associated with faster operating times when comparing similar experience levels and less adverse outcomes.^23^

One of the limiting factors in microsurgery and supermicrosurgery is the high demand for dexterity and stability of the hands, and the technique usually takes several years for a surgeon to master. A vessel size of 2 mm was chosen for time and resource purposes. We recognize this is only "micro" and not "supermicro" in terms of size but we find it reasonable to believe that the impact of robot use would be more significant for supermicrosurgical vessel (0.3-0.8 mm) anastomosis.

Moreover, some surgeons may experience more physiological tremors with age, potentially limiting the microsurgeons’ ability to perform complex procedures. The surgical fields are often small and located in parts of the body that are hard to reach, which adds difficulty and physical demand on the surgeon. Studies regarding ergonomics have shown that the neck and back are the common problematic areas for microsurgeons. Musculoskeletal issues arise from working in a fixed position for a long time under the microscope where movements and body position are limited during surgery.^24^ Robotic surgery has an advantage in ergonomically difficult surgical situations.^25^ Learning robot-assisted techniques alongside the traditional manual techniques may make it possible for surgeons to perform more complex procedures earlier in their training or career. There is also a chance of prolonging their surgical careers by mitigating musculoskeletal issues. Additionally, robot-assisted microsurgery could open opportunities to offer treatment to patients who are not currently considered as candidates for surgery owing to their vessel size.

A common disadvantage of robotic microsurgery is the lack of tactile or haptic feedback.^21^^,^^25^ Surgeons depend on the sensation in their hands to know how hard they have to pull the thread while tying the knots. The experts in our study reported they had to rely on their eyes, not their hands, and therefore were not dependent on haptic feedback. Although it is difficult to measure precisely how the experience makes a difference, in this aspect, these comments from the participants suggest the need for further investigation.

To our knowledge, our study population is the largest to date regarding learning curve studies on robotic microsurgery.^13^^,^^19^, ^20^, ^21^, ^22^ All participants received the same written information and performed the same introductory exercises; however, the same person was not always instructing them. Thus, our methods’ limitations include the amount of feedback and assistance that potentially differed between instructors, for example in how the needle was presented in the robot or how well the assistant flipped the vessels, which could expedite the task. The study investigators observed that the assistant was not as accommodating for the expert group as for the intermediate or novice surgeons. The expert group adjusted the needle by themselves, but the intermediate and novice always received the needle presented in the same way to make it easier. Identifying this early in the study, the same assistant was used for every session to eliminate the assistant as a confounding variable.

Another variable that may account for differences initially between the groups was the ability to perform small/precise movements with the instruments. The expert made smaller, delicate movements while performing the anastomosis manually and with robotic assistance. One of the limiting factors with MUSA-2 is the range of motion; larger movements cause the instruments to move into an end position where the surgeons could no longer control the robot and it had to be repositioned manually. During the practice exercises before performing their first robotic-assisted anastomosis, the novice and intermediate surgeons demonstrated considerably large movements. However, after a few sessions, their movements became increasingly smaller, but never as delicate as those of the surgeons in the expert group. Additionally, the robot assistance helped the novice group perform faster anastomosis without making more errors.

One major factor influencing the performance included time between sessions. The surgeons whose sessions were closer in time had a steeper decline in their time to perform an anastomosis than those who had longer intervals between their sessions. The quality of the anastomosis was also affected by the number of days that had passed since the previous session. Other studies eliminated this factor by having the sessions every day at the same time.^26^ This was impossible for us to arrange owing to the work hour constraints of the participants. Notably, in the robotic results, we chose to sample the second session instead of the first due to the high number of participants reaching the 60-minute time cap during the first session, which we considered as outliers.

The surgeon's personality and its impact on surgical performance is the most challenging confounder to measure. In the current study, each participant had different coping strategies when learning new skills. Additionally, only some participants expressed a personal interest in learning robotic microsurgery. Regarding learning curve evaluation, personality contribution to performance is scarcely mentioned, but from an observer's qualitative perspective, it can affect the result. Twin studies suggest that motor skill acquisition is influenced mainly by genetics in the early phase of skill acquisition, which then trends toward environmental factors with practice.^27^

Future directions of robotics are looking toward integrating artificial intelligence (AI). In most robot-assisted surgery, the platforms are only semiautonomous, meaning the programs operate on supervised learning. AI assistance has allowed the operator experience to be enhanced by decreasing tremors and fatigue. As the robot interrupts the haptic feedback experienced by the surgeon, biosensors are currently being developed to mimic the mechanical and haptic feedbacks to the surgeon. These technologies could revolutionize how surgery is performed in the future. However, the transition to a fully automized robot that performs the surgery, making unsupervised, irreversible decisions comes with severe ethical and legal concerns.^1^ Some centers have employed robotics to assist with the harvest of deep inferior epigastric perforator flaps, hypothesizing that this will minimize abdominal morbidity.^28^^,^^29^ There are additional financial costs associated with the implementation of a robotic system in a microsurgical setting. However, a vital part of our job, for the advancement of our specialties and patient's benefit, is to set up new techniques and safer ways of performing the established ones. Consequently, there are associated fiscal and time costs, that can easily be overcome with patience and a reasonable amount of cases.^30^

## Conclusion

Our study indicates similar learning curves for robot-assisted anastomosis among participants with varied previous microsurgical experiences. Experts of microsurgery performed manual anastomosis faster and better technically than the other groups. However, the differences in using robotic assistance were not as diverse among the groups. In conclusion, our results suggest similarities in learning curves for robot-assisted anastomosis among surgeons with varied experience levels; experts performed better technically than the other groups in manual anastomoses throughout the study but the robot helped novice and intermediate surgeons perform similarly to the expert group.

## Conflict of interest statement

The Corresponding Author has been part of the Medical Advisory Board of Microsure since June 2022. All other authors have no financial disclosures or conflicts of interest.

## Appendix Supplementary materials

Image, video 1

Image, video 2

Image, video 3

Image, video 4

Image, video 5

Image, video 6

Image, video 7

Image, video 8

Image, video 9

Image, video 10

Image, video 11

Image, video 12
